# Chromatin Remodeling Pathways in Smooth Muscle Cell Differentiation, and Evidence for an Integral Role for p300

**DOI:** 10.1371/journal.pone.0014301

**Published:** 2010-12-13

**Authors:** Joshua M. Spin, Thomas Quertermous, Philip S. Tsao

**Affiliations:** Division of Cardiovascular Medicine, Department of Medicine, Stanford University School of Medicine, Stanford, California, United States of America; Brunel University, United Kingdom

## Abstract

**Background:**

Phenotypic alteration of vascular smooth muscle cells (SMC) in response to injury or inflammation is an essential component of vascular disease. Evidence suggests that this process is dependent on epigenetic regulatory processes. P300, a histone acetyltransferase (HAT), activates crucial muscle-specific promoters in terminal (non-SMC) myocyte differentiation, and may be essential to SMC modulation as well.

**Results:**

We performed a subanalysis examining transcriptional time-course microarray data obtained using the A404 model of SMC differentiation. Numerous chromatin remodeling genes (up to 62% of such genes on our array platform) showed significant regulation during differentiation. Members of several chromatin-remodeling families demonstrated involvement, including factors instrumental in histone modification, chromatin assembly-disassembly and DNA silencing, suggesting complex, multi-level systemic epigenetic regulation. Further, trichostatin A, a histone deacetylase inhibitor, accelerated expression of SMC differentiation markers in this model. Ontology analysis indicated a high degree of p300 involvement in SMC differentiation, with 60.7% of the known p300 interactome showing significant expression changes. Knockdown of p300 expression accelerated SMC differentiation in A404 cells and human SMCs, while inhibition of p300 HAT activity blunted SMC differentiation. The results suggest a central but complex role for p300 in SMC phenotypic modulation.

**Conclusions:**

Our results support the hypothesis that chromatin remodeling is important for SMC phenotypic switching, and detail wide-ranging involvement of several epigenetic modification families. Additionally, the transcriptional coactivator p300 may be partially degraded during SMC differentiation, leaving an activated subpopulation with increased HAT activity and SMC differentiation-gene specificity.

## Introduction

The ability of mature vascular smooth muscle cells (SMC) to modulate their phenotype is responsible in large part for many of the specific manifestations and genesis of vascular diseases such as hypertension, atherosclerosis, and post-angioplasty restenosis [1], [2], [3]. Unlike skeletal and cardiac myocytes, vascular SMCs respond to environmental cues by de-differentiating: down-regulating SMC marker and contractile proteins (e.g. smooth muscle [SM] α-actin, transgelin, SM-myosin heavy chain [MHC]), migrating into the neointima, proliferating, and secreting matrix and remodeling factors.

The molecular mechanisms underlying lineage determination and terminal differentiation of these cells have received much attention, but the genetic programs that regulate these processes have not been fully defined. Experiments have identified levels of epigenetic regulation underlying SMC plasticity, including specific histone modifications that appear to support the SMC lineage and alter the ability of the transcriptional regulator serum response factor (SRF) to target SMC marker-gene promoters [4], [5].

DNA in eukaryotic nuclei is packaged into repeating units of chromatin, composed of nucleosomes, with 145–147 DNA base pairs wrapped around an octameric core containing two molecules each of histones H2A, H2B, H3 and H4. The core is then stabilized into higher order structures by linker histone H1 [6], [7], [8]. The amino-terminal portions of core histones contain flexible protease-sensitive tails which are evolutionarily conserved sites for post-translational modifications, including methylation, acetylation, phosphorylation, ubiquitylation, and ADP-ribosylation [9], [10], [11]. These modifications are correlated with replication, chromatin assembly, and transcription [7], [12]. In general, acetylation of histones is transcriptionally activating, while mono-, di- and tri-methylation may cause silencing or activation depending on which particular lysine residues are modified.

Various enzymes and enzymatic complexes (e.g. histone acetyltransferases (HATs), histone deacetylases (HDACs), histone and DNA methyltransferases (HMTs and DNMTs) regulate transcription through chromatin modification, partly through a “histone code” in which combinations of specific residue modifications regulate unique biological outcomes [13]. Numerous proteins and protein complexes have been identified that may “read” the code and recruit transcription factors or repressors.

HATs are well characterized covalent histone modification systems, and consist of several protein families including CBP/p300 [7]. CBP and p300 are paralogues, and act as multifunctional transcriptional co-activators involved in such varied processes as embryonic development, differentiation, proliferation and apoptosis [14]. Expressed ubiquitously during mouse development, they interact with numerous transcription factors, integrating complex signal transduction pathways at the level of gene transcription. CBP and p300 are necessary factors in skeletal myogenesis and cardiomyogenesis [15]–[19]. Several studies have indicated that p300 may be necessary for SMC differentiation, and is likely also essential for phenotypic switching [4], [20]–[25].

The P19-derived A404 embryonal cell line differentiates toward the SMC lineage in the presence of retinoic acid (RA), and allows selection for cells adopting a SMC fate through a differentiation-specific drug marker (puromycin) [20]. We previously performed expression profiling of differentiating A404 cells, and identified numerous ontology-based pathways that undergo differential regulation [26]. Among the most prominent pathways identified was chromatin remodeling. We sought to further characterize the role of chromatin remodeling and p300 in SMC differentiation. Results support the hypothesis that chromatin remodeling factors, and p300 in particular, are important for SMC phenotypic switching.

## Methods

### Cell culture, RNA isolation

The A404 cell culture protocols, microarray hybridization, and RNA isolation methods were previously published [26]. Briefly, undifferentiated P19-A404 cells (Control) were treated with 1 µmol/L all-*trans* retinoic acid (RA) for 24 hours (RA24), 48 hours (RA48), 96 hours (RA96), or 96 hours followed by puromycin (Puro). RNA was harvested from multiple replicates at each of the four time points, isolated by chloroform extraction followed by Qiagen RNeasy Midi Kit Protocol, and quantitated by Nanodrop (Agilent Technologies, Santa Clara, CA).

Human coronary SMCs (Clonetics, Lonza Group Ltd., Switzerland) between passages 3 and 6 were propagated in SMGM – growth medium (Clonetics) to 85% confluence (subset harvested as control) and then placed in serum-free SMBM – basal medium (Clonetics) for either 48 or 72 hours, with a minimum of three plates per sample per time point. Cells were then harvested for protein or RNA isolation as described.

### HDAC inhibition with trichostatin A

Culture protocols were identical to those used previously, except that A404 cells were treated instead for 48 hours with vehicle - diluted 100% ethanol (Control), with 10 ng/mL of trichostatin A (TSA) (Sigma, St. Louis, MO), with vehicle +1 µmol/L all-*trans* retinoic acid (RA) (Sigma), or with trichostatin and retinoic acid (TSA/RA). Protocol was derived from Minucci, et al [27]. Multiple replicates and multiple plates/replicate were utilized. RNA was harvested, isolated, and quantitated as above. A separate set of replicates were treated identically, but instead harvested for histone extraction using established methodology (below) [28].

### Chemical p300 HAT inhibition

Lys-CoA-TAT, a cell-permeable chemical inhibitor of p300 HAT, was the kind gift of Philip A. Cole and colleagues at Johns Hopkins [29]. A404 cells (± RA and ±TSA) were treated for 24 or 48 hours with 20 µM Lys-CoA-TAT, using an empty TAT-peptide as a control. They were then harvested at time 0, and at 48 h for RNA or 24 h for protein. All medium was replenished at 24 hours. All treatment wells were >90% confluent at time of harvest.

### siRNA knockdown of EP300/Ep300

Cultured human SMC and A404 cells were transfected with Silencer Select siRNAs targeting either EP300 or Ep300 transcripts as appropriate, as well as GAPDH (glyceraldehyde 3-phosphate dehydrogenase) as a positive control, and scrambled negative control siRNA (Applied Biosystems). Forward transfection was performed in 6-well plates using 3 µL/well of Lipofectamine RNAiMAX (Invitrogen) per recommended protocol, using 10 nM (30 pmol) siRNA, when cells were 40–50% confluent. Three replicates per condition were included. Human SMCs were serum starved for 48 hours, beginning 24 hours after transfection. A404 cells were treated with DMSO vehicle or 1 µmol/L all-*trans* retinoic acid (RA) for 24 or 48 hours, starting 5 hours post-transfection. Cells were then harvested for RNA.

### Histone and total protein isolation

For the HDAC inhibition studies, cells were collected, washed with ice-cold PBS, and centrifuged for 5 minutes at 600×g. The pellet was re-suspended in ice-cold lysis buffer, centrifuged, and washed with 10 mM Tris-HCl, 13 mM EDTA. After spinning again, the pellet was re-suspended in 0.4 N H_2_SO_4_ and acid extracted on ice for 1 hour. Centrifugation at 10,000×g was followed by acetone precipitation at −20°C for 1 hour, and centrifuging at 10,000×g. The histone pellet was air-dried, re-suspended in water and stored at −80°C. For some studies, histones were isolated using the Active Motif Histone Purification Mini Kit (Carlsbad, CA) per manufacturer's protocol. Protein was quantitated by either Thermo Scientific BCA Protein Assay Kit (Rockford, Ill) or Bio-Rad DC Protein Assay (Hercules, CA) with a bovine serum albumin standard (R^2^ = 0.999).

Isolation of total protein for p300 immunoblotting was performed by rinsing with ice-cold PBS, lysing the cells with CelLytic-M (Sigma) containing protease inhibitor cocktail (Sigma), spinning at 15,000×g for 15 min and collecting the supernatant. Protein was quantitated by DC protein assay.

### Western blots

Western immunoblotting of HDAC inhibition samples was performed using 1–4 µg/lane protein in Tris-Glycine SDS Sample Buffer (Invitrogen, Carlsbad, CA) loaded onto 12% or 15% SDS-PAGE gels. Gapdh was used as a loading control. Protein was transferred to PVDF (polyvinylidene fluoride) membranes, blocked with 3% PBS-milk (Upstate Cell Signaling Solutions, Waltham, MA), and probed with 1 µg/mL rabbit anti-acetyl histone H4 or 0.5–1.0 µg/mL anti-histone H4 (Upstate) followed by 0.2 ng/mL goat anti-rabbit horseradish-peroxidase-conjugated IgG, and developed with Visualizer Working Solution (Upstate). Membranes were exposed using BioMax MS Film (Kodak, Rochester, NY).

Western blots of human coronary SMC and A404 p300 utilized 1 µg/mL rabbit anti-p300 (N-15, Santa Cruz Biotechnology, Inc, Santa Cruz CA). Samples were diluted in NuPAGE LDS 4× Sample Buffer (Invitrogen) and loaded onto NuPAGE Novex 3–8% Tris-Acetate gels with SeeBlue Plus2 Ladder for size calibration. Other processing was similar to that described above. Exposed films were scanned, and integrated band densities were obtained and normalized to Gapdh and background using ImageJ [30]. Mean ± std dev of lane duplicates are shown in density units.

### Quantitative reverse-transcription PCR

Total RNA was converted to cDNA using MMLV reverse transcriptase. The cDNA was amplified in triplicate on the ABI PRISM 7900HT with Taqman primers and probes (Applied Biosystems, Foster City, CA). Gene expression levels were normalized to corresponding 18S internal controls. At least two representative samples from each time point were evaluated. Fold changes were calculated by the method of ΔΔC_t_.

### Data analysis

Microarray methods have been previously described [26]. All microarray data were submitted to the Gene Expression Omnibus (GEO) database at the NCBI (GSE1506; http://www.ncbi.nlm.nih.gov/geo/). Array data were probed using GOMiner [31] and the Database for Annotation, Visualization and Integrated Discovery (DAVID) [32] to identify all genes annotated with ontology terms related to chromatin remodeling and assembly, gene silencing, or histone modification. These were then cross-referenced against genes showing significant regulation (false discovery rate - FDR<1) during A404 SMC differentiation. Relative overabundance calculations were performed using Fisher's Exact Test. Heatmaps were created using Heatmap Builder 1.0 (Ashley E., Spin J., Watt C., Stanford) [26]. Two-tailed unpaired t-tests were used to evaluate significance for qRT-PCR results.

The p300-transcription factor interactome was derived with PathwayAssist 3.0 (Ariadne Genomics, Inc., Rockville, MD), which utilizes KEGG, DIP, and BIND databases and natural language scans of PubMed to define connectivity among genes to delineate a functionally related network.

## Results

### Chromatin remodeling genes show widespread regulation with *in vitro* SMC differentiation

In previously published work, A404 P19 mouse embryonal carcinoma cells were induced to differentiate into SMCs using all-trans retinoic acid (RA) treatment for 96 hours, followed by 48 hours of puromycin (eliminating remaining cells not expressing SM α-actin), and then RNA-harvested for microarray transcriptional profiling [26]. We used a 60-mer microarray platform (Agilent, Palo Alto, CA) with 20,280 mouse transcripts derived from the National Institute on Aging clone set, and identified 2,739 genes that were significantly upregulated from untreated cells after differentiation was completed (FDR<1), as well as 2,227 downregulated genes. Temporal patterns of regulation were also identified.

**Figure 1:**
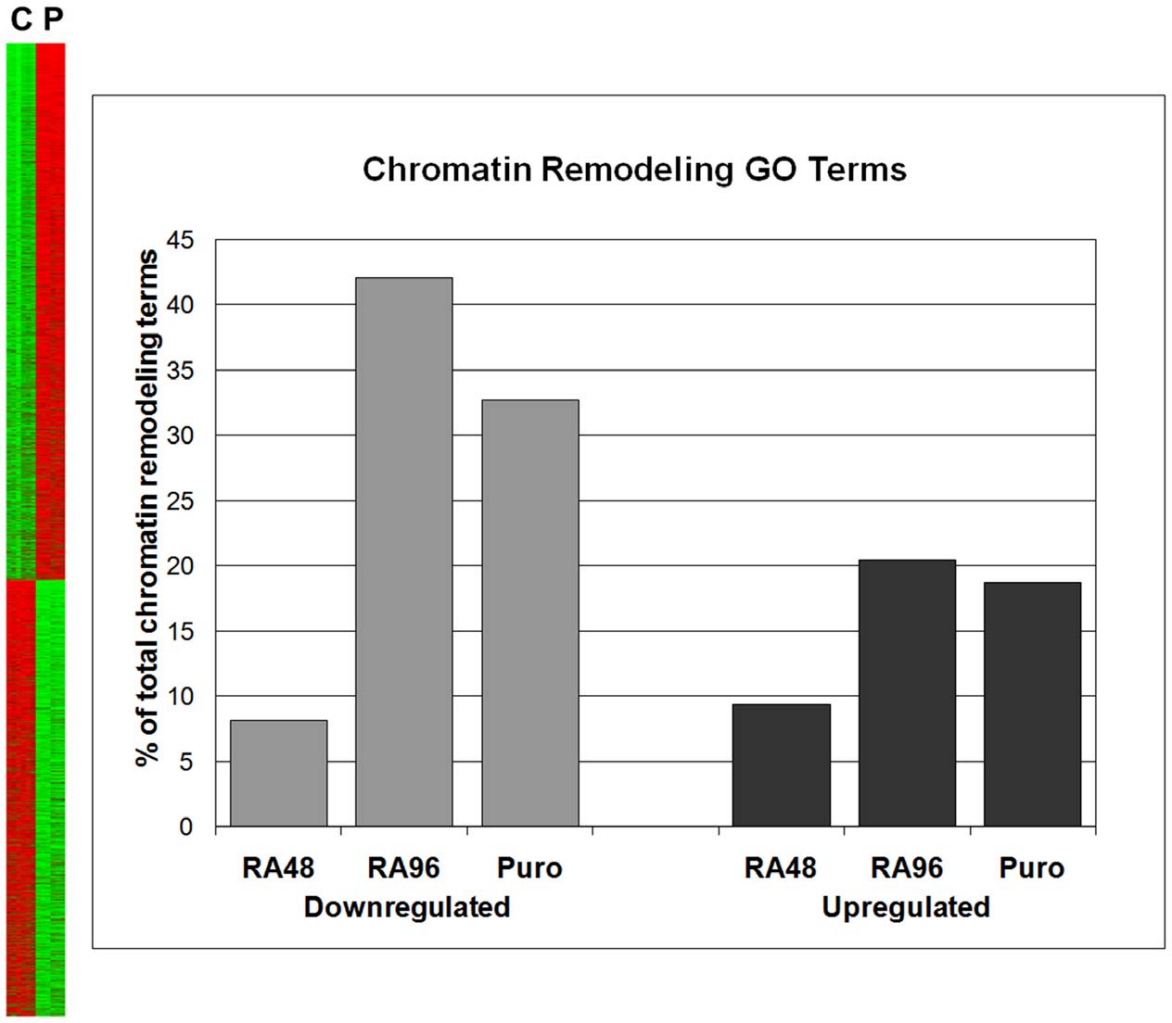
Heatmap and regulated chromatin remodeling genes. **Left**: Row-normalized gene expression heatmap of A404 cells treated with all-trans retinoic acid for 96 hours, then puromycin for 48 hours, significant at FDR<1. Shown are 2,739 genes upregulated (P>C), and 2,227 genes downregulated (C>P). C = Control replicates, n = 6. P = Puromycin group replicates, n = 6. Green: down. Red: up. One gene/row. **Right**: Regulated chromatin remodeling genes during A404 differentiation. GO annotation terms for selected pairwise SAM comparisons (FDR<1) of treatment groups were obtained, using gene lists with unique names. Values are % of total set of 171 chromatin remodeling genes on array. Downregulated and Upregulated  =  vs. control A404 cells. RA48 and RA96  =  RA-treated for 48 or 96 h. Puro  =  RA-treated 96 h, then puromycin-treated for 48 h.

In an updated analysis, ontologic pathways within these data were scrutinized using GOMiner and DAVID. The array was found to contain 171 unique genes related to chromatin remodeling, including such aspects as histone tagging (acetylation, deacetylation, methylation), chromatin assembly/disassembly, and silencing. A substantial percentage of these genes showed significant regulation (FDR<1) during SMC differentiation, with more downward regulation than upward overall (**Figure 1**). The smallest changes were seen early in differentiation (8.2% down and 9.4% up at 48 hours of RA), while the largest response occurred in the mid-portion of the time-course (42.1% down and 20.5% up in cells treated 96 h with RA vs. control). After using puromycin, 32.7% and 18.7% of chromatin remodeling genes remained down- and upregulated respectively.

### Pathway analysis of chromatin remodeling genes during SMC differentiation shows diverse response

**Figure 2:**
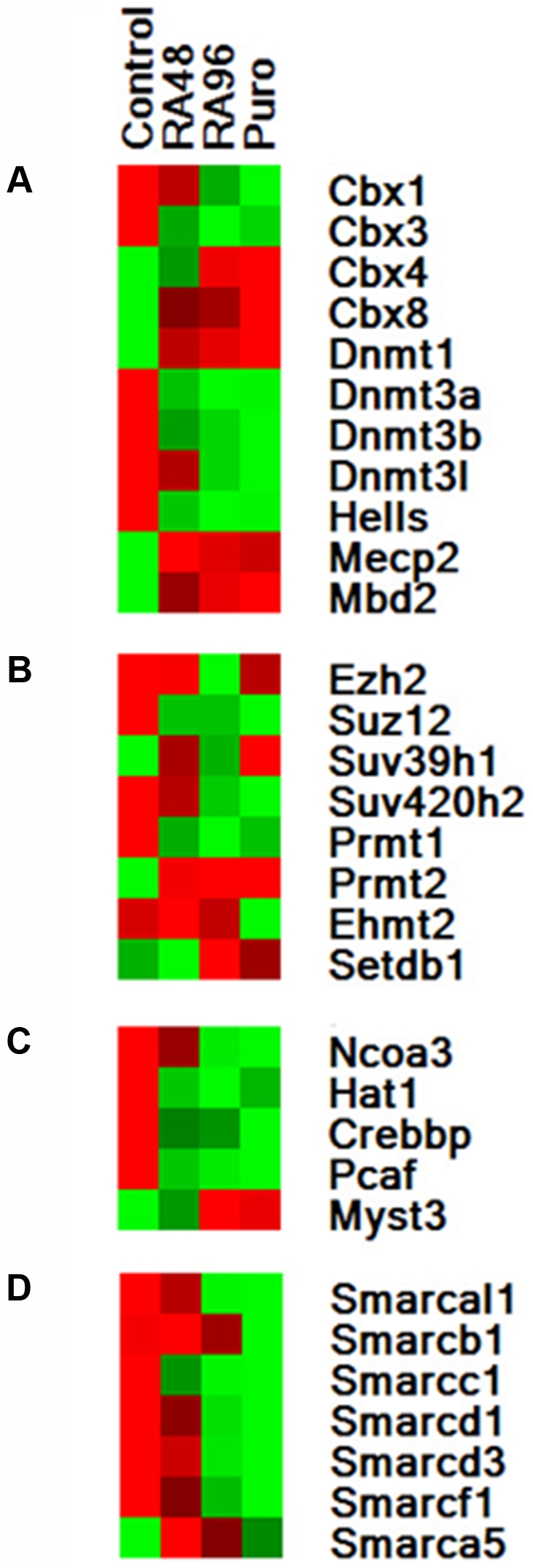
Differential regulation of selected chromatin remodeling gene classes with SMC differentiation. **A**. DNA methyltransferases. **B**. Histone methyltransferases. **C**. Histone acetyltransferases. **D**. SWI/SNF family. Row-normalized heatmaps are shown labeled with gene symbols. Control  =  untreated A404 cells. RA48/RA96  =  Retinoic acid treated for 48 and 96 h respectively. Puro  =  Treated with RA for 96 hours, then puromycin for 48 hours. Green: down. Red: up.

Seeing the extent of chromatin-related gene regulation, we next inspected the specific biological/molecular pathways within the set, and found a variety of patterns within individual gene classes. One highly represented class included proteins that silence chromatin via DNA methylation (**Figure 2a**), and included chromobox genes, members of the DNMT protein family, as well as methyl-CpG binding domain (MBD) family genes.

Another well-represented class included histone methyltransferases (HMTs). Generally speaking, methylated histones bind DNA more tightly, which inhibits transcription (**Figure 2b**).

Several known HAT genes displayed significant regulation (**Figure 2c**) during SMC differentiation. Some were downregulated, including Ncoa3, Hat1, CBP/Crebbp, and Pcaf. In contrast, Myst3 was upregulated by 96 h and remained so. While p300/Ep300 was not on the array, qRT-PCR Taqman studies showed minor gene upregulation with A404 differentiation over control: mean 1.2-fold (24 h, RA), 1.6-fold (48 h RA), and 1.3-fold (96 h, RA). Although p300 transcription changes were small, it should be noted that numerous signaling pathways are known to post-transcriptionally regulate its protein activity.

A number of regulated genes contained SWI/SNF protein subunits, which are involved in chromatin assembly and remodeling and are characterized by DNA-dependent ATPase activity. Nearly all of these genes were decreased during the treatment course (**Figure 2d**). Smarcal1, Smarcb1, Smarcc1, Smarcd1, and Smarcd3, and Smarcf1 all showed significant downregulation by 96 h of RA treatment and remained so. However, Smarca5 was strongly upregulated early. Transcription then tapered downward, but remained significantly increased throughout.

A404 SMC differentiation was also accompanied by highly variable regulation of histone deacetylases (**Table 1**). Two Class I HDACs, Hdac2 and 3, showed minimal to no significant change until the final selection step with puromycin, and then moved in opposite directions. Hdac1, in contrast, was downregulated by 96 h of RA-treatment and remained so. The Class II HDACs, which are thought to be more tissue specific, displayed dramatically different responses from each other. For example, Hdac6 was downregulated 50-fold by 96 h, while Hdac7a and Hdac9 both showed significant upregulation. Notably, Class III HDAC Sirtuin 1 (Sirt1), a protein- and histone-deacetylase known for inhibiting differentiation of skeletal muscle via its involvement in Pcaf and p300 suppression [33], [34], showed early and persistent downregulation throughout. Another member of the same family, Sirt3, was also significantly downregulated at 96 h.

**Table 1:** HDAC expression with SMC differentiation.

Probe	Retinoic Acid-96 hours	Puromycin
SM α-actin	180.11	234.03
SM-MHC	25.98	84.00
Hdac1	0.58	0.47
Hdac2	0.81	1.38
Hdac3	NS	0.16
Hdac5	NS	0.13
Hdac6	0.02	0.02
Hdac7a	3.75	8.67
Hdac9	4.93	14.32
Sirt1	0.34	0.44
Sirt3	0.67	NS

Fold changes vs. control for histone deacetylase genes and SMC marker genes during A404 differentiation time-course. NS: not significant. For significant changes, FDR<1.

### HDAC inhibition accelerates rate of SMC marker gene increase with differentiation

**Figure 3:**
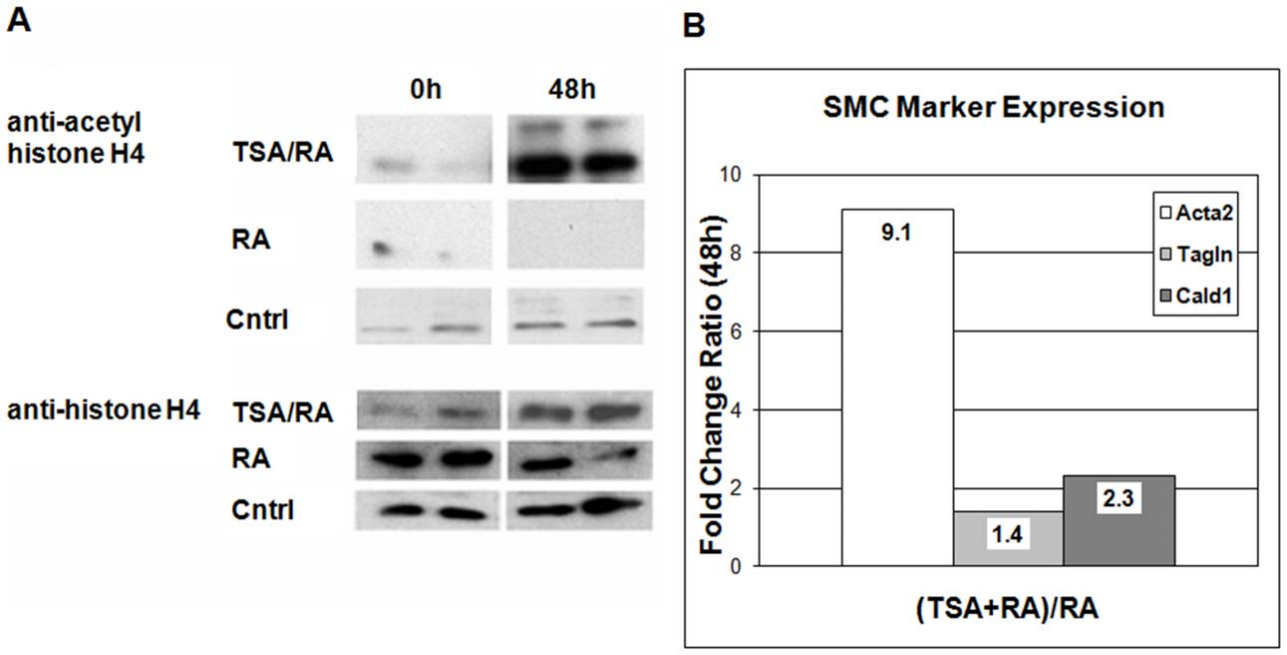
Trichostatin A and SMC differentiation markers. **A**. Western blots of A404 cell histone extract, using anti-acetyl histone H4 or anti-histone H4 primary antibody. Cntrl  =  Control; RA  =  retinoic acid; TSA/RA  =  trichostatin A+ retinoic acid. Time 0 and 48 h of treatment are shown. **B**. Increased relative expression of SMC differentiation markers after 48 h in TSA + RA-treated A404 cells vs. RA-treated alone. For all ratios, p<0.05. Acta2  =  SM α-actin, Tagln  =  transgelin/SM22α, Cald1  =  caldesmon 1.

Given the varying regulation of various HDAC family members, we investigated the effect of HDAC suppression using Trichostatin A (TSA) during RA-induced SMC differentiation. Immunoblotting revealed some baseline histone H4 acetylation present in proliferating, untreated A404 cells (**Figure 3a**). Treatment with TSA and RA together led to greatly increased levels of acetyl-histone H4 compared with RA alone or untreated control, verifying that TSA at this concentration inhibits histone deacetylation in the A404 model. Control H4 histone levels did not alter significantly with RA treatment, with time, or with the addition of TSA.

As expected, treatment with retinoic acid significantly increased expression of the SMC markers SM α-actin (Acta2), transgelin/SM22α (Tagln), and caldesmon 1 (Cald1) markers at 48 hours relative to vehicle-treated cells: 22.0-fold, 3.2-fold, and 4.1-fold respectively (FDR<1, microarray). However, treatment with TSA combined with RA further increased expression for all three markers compared with RA alone by qRT-PCR (**Figure 3b**). Ratios of TSA-RA-treated expression to RA-treated were 9.1-fold (Acta2), 1.4-fold (Tagln), and 2.3-fold (Cald1).

### SMC differentiation is accompanied by widespread regulation of the p300 transcription factor-interactome

**Figure 4:**
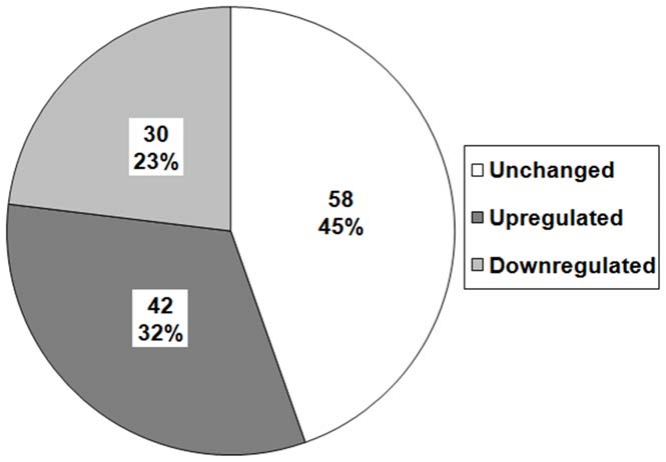
p300 interactome transcription factors. Transcription factors in the p300 interactome which demonstrated significant regulation during A404 differentiation vs. untreated cells (FDR<1). White  =  unchanged. Light grey  =  upregulated. Dark grey  =  downregulated. Gene number and % of interactome shown for each section.

P300/Ep300 is believed to be an important regulator of smooth muscle-specific gene expression, and is a known key player in epigenetic regulation during skeletal and cardiac myocyte differentiation. PathwayAssist, a literature-based program for mapping molecular relationships, was used to identify all transcription factors known to interact directly with p300/Ep300. This yielded a total of 162 genes, of which 130 were on the array (**Figure 4**). During SMC differentiation of A404 cells, 72 (55.4%) of these genes showed significant differential regulation, pathway over-representation that was highly significant by Fisher's Exact Test (p<0.0001). From this set, 42 (58.3%) were up and 30 (41.7%) were down. This supports the idea that p300 may be central to SMC differentiation, although it does not specify the precise nature of its involvement.

### P300 protein levels decrease with SMC differentiation

**Figure 5:**
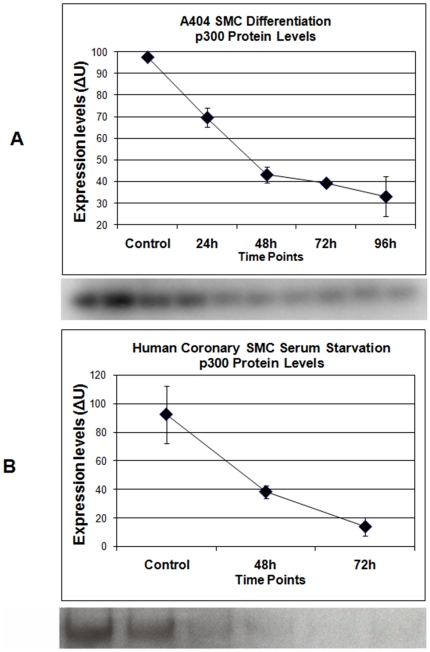
p300 protein levels with SMC differentiation/serum-starvation. Plots of scanned, digitized Western blots probed with anti-p300, and representative blots. **A**. Top: A404 cells treated with retinoic acid for up to 96 hours. Bottom: A404 p300 Western blot. Two lanes per time point, same order as plot. **B**. Top: primary human coronary artery SMCs, serum starved for up to 72 hours. Bottom: CASMC p300 Western blot. Two lanes per time point, same order as plot. Integrated band densities were normalized to Gapdh and background. Mean ± standard deviation of lane duplicates are shown in density units.

Having established that minimal transcriptional regulation of p300 accompanies SMC differentiation, but that the majority of the p300 interactome shows significant change, we next used Western blotting to examine p300 protein levels. P300 protein levels decreased dramatically during the 96 hour retinoic acid time-course (**Figure 5a**). Nearly all of this change occurred within the first 48 h of treatment.

**Figure 6:**
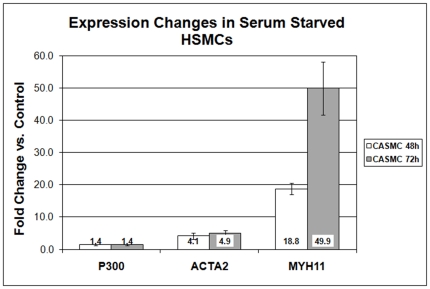
Gene regulation in human coronary SMCs by qRT-PCR. Cells were serum starved in basal medium for up to 72 h to induce differentiation. P300  =  EP300 HAT, ACTA2  =  SM α-actin, MYH11  =  SM-MHC. 48 and 72 h time points are shown as fold change from baseline ± standard error. All values are significantly increased vs. control (p<0.05).

We sought to confirm this finding in another model of SMC differentiation. Primary vascular smooth muscle cells rapidly de-differentiate *in vitro*, a process that partially reverses under conditions of serum-starvation [2]. Human coronary smooth muscle cells were serum starved in basal medium for 48 and 72 hours and then harvested for RNA or protein. Taqman qRT-PCR showed the expected increases in ACTA2 and MYH11 (SM-MHC) with starvation, while p300 showed similar regulation to A404 differentiation (a mild 1.4-fold increase) (**Figure 6**). Western blotting results paralleled those in A404 cells, with p300 levels decreasing substantially during the time course (**Figure 5b**).

### Direct chemical inhibition of p300 histone acetyltransferase activity blunts SMC differentiation

Because p300 possesses other functions beyond acetyltransferase activity, we sought to confirm that p300's HAT activity is important in SMC differentiation. Several small molecule chemical inhibitors have been developed which act as bisubstrate analogs [35], [36], [37]. A potent and selective cell-permeable inhibitor of p300 HAT activity, Lys-CoA-TAT, was previously developed by Philip A. Cole and colleagues[29].

**Figure 7:**
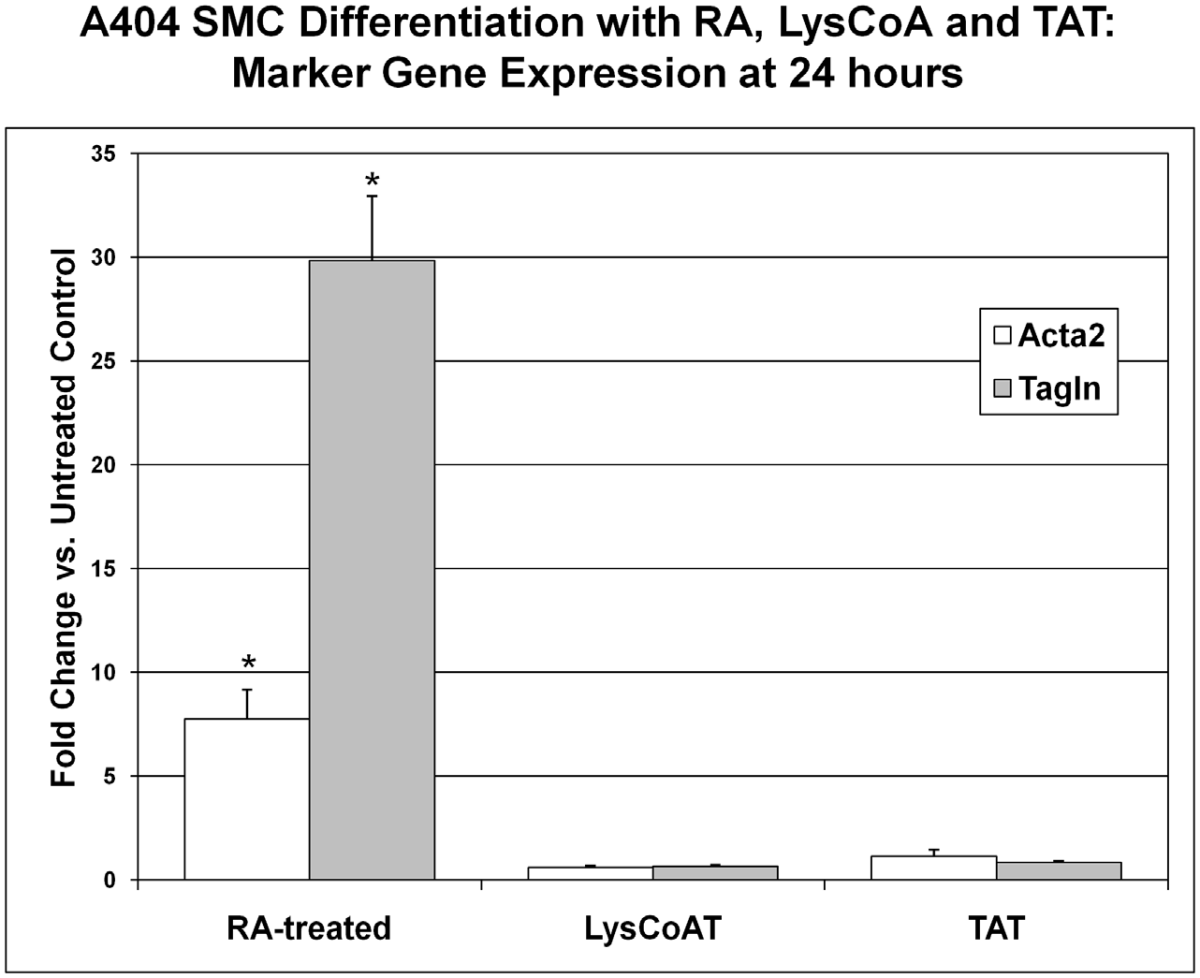
p300 HAT inhibition controls. A404 cells were treated for 24 hours with: 1) retinoic acid (RA), 2) 20 µM Lys-CoA-TAT (LysCoAT), or 3) 20 µM control TAT peptide (TAT). Expression levels obtained by qRT-PCR for SMC markers are shown as fold change from baseline untreated cells ± standard error. *  =  significantly (p<0.05) different from control.

**Figure 8:**
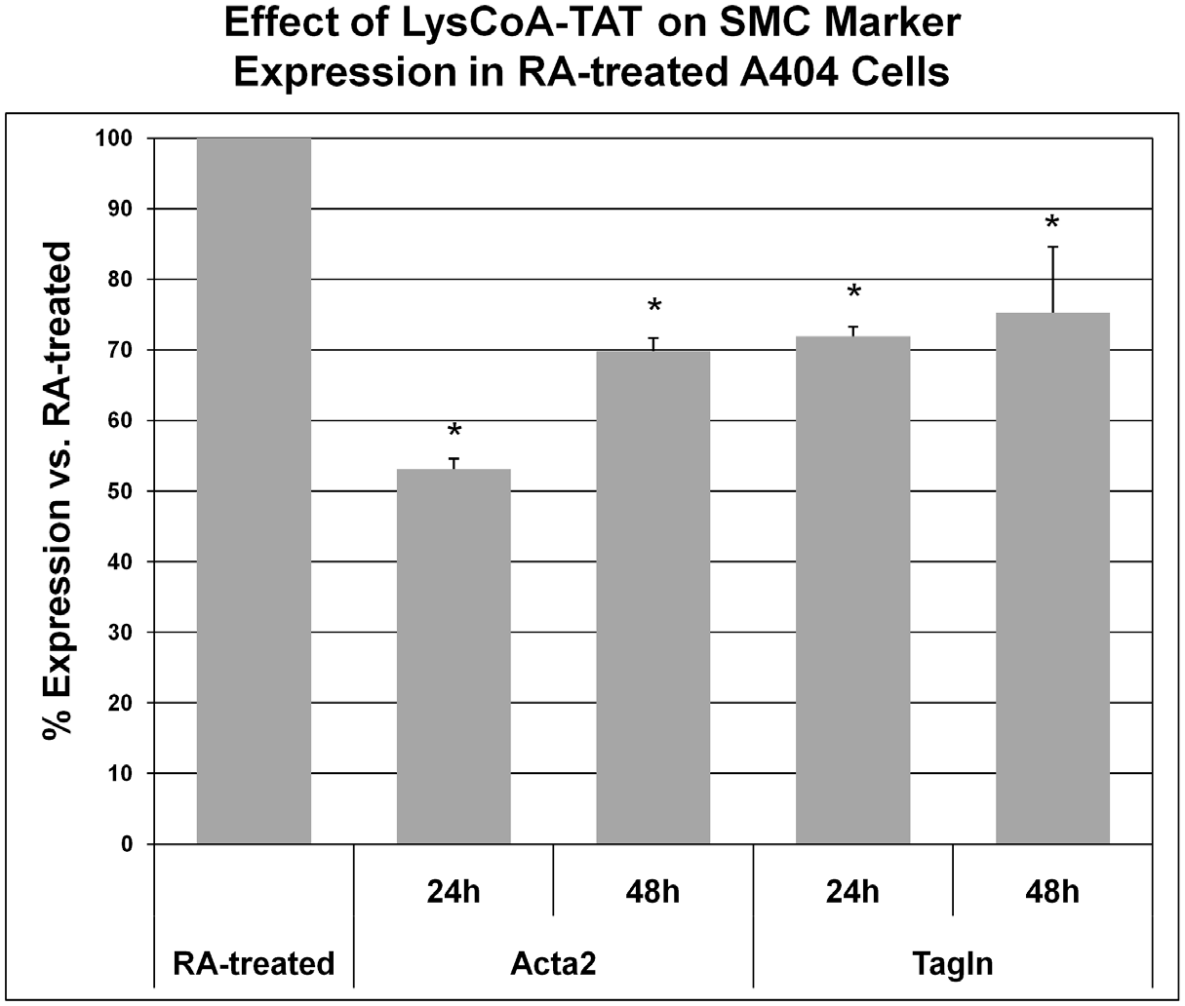
p300 HAT inhibition and A404 SMC differentiation markers. A404 cells were treated for 24 or 48 hours with retinoic acid (RA) ±20 µM Lys-CoA-TAT. Shown are qRT-PCR expression values for Acta2 and Tagln, calculated as a percent of the RA-induced marker level for that time point. An RA-treatment bar is shown for comparison. *  =  significantly (p<0.05) different from cells treated with RA alone.

**Figure 9:**
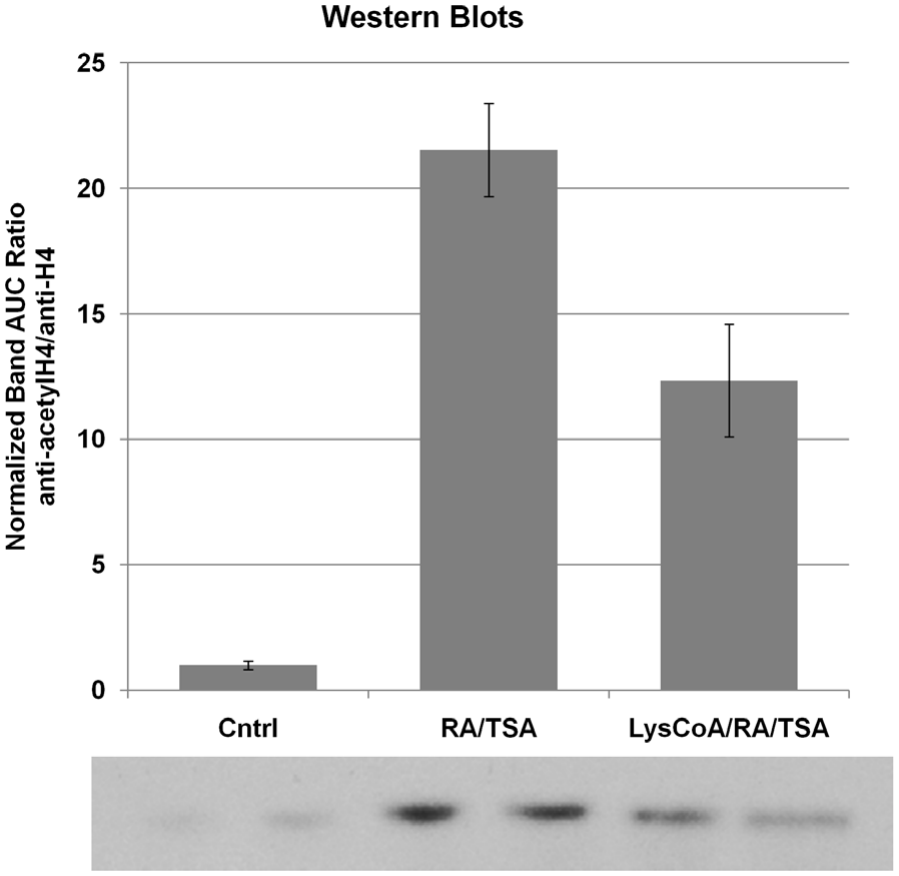
p300 HAT inhibition and trichostatin A. Area-under-the-curve (AUC) pixel densitometry was calculated for scanned, digitized anti-acetyl-histone H4 Western blots (shown at bottom) of A404 cells either untreated (Cntrl), or treated with trichostatin A+ retinoic acid (RA/TSA) or trichostatin A+ retinoic acid + Lys-CoA-TAT (LysCoA/RA/TSA) for 24 hours. Values shown are a ratio of anti-acetyl-histone H4:anti-histone H4, normalized to untreated control AUC ± standard error for three experiments. Western lanes (in duplicate) correspond to graph columns.

A404 cells (± retinoic acid) were treated over 24 or 48 hours with 20 µM Lys-CoA-TAT, using an empty TAT-peptide as a control. Neither Lys-CoA-TAT nor TAT-peptide alone had any significant impact on A404 cell expression of SMC markers by qRT-PCR. (**Figure 7**). At both time points Lys-CoA-TAT treatment significantly (p<0.05) decreased the effects of retinoic acid on SMC marker expression (**Figure 8**). Suppression was 46.9% for Acta2 and 28.0% for Tagln at 24 hours. Further, co-incubation of A404 cells with TSA, RA and Lys-CoA-TAT (**Figure 9**) confirmed that the HAT inhibitor was effective at the dose used, as it successfully blunted the increase in TSA-induced histone H4 acetylation seen with RA-induced SMC differentiation by 43%.

### Knockdown of p300 with siRNA accelerates SMC differentiation

Since p300 protein levels decreased during SMC differentiation, we also investigated the impact of EP300/Ep300 knockdown using siRNA in both the human SMC and A404 models. The transfection protocol used produced an average 95.4% reduction in expression of the GAPDH positive control. In human SMCs, EP300 expression was decreased 66.8% in serum-fed cells and 75.7% in cells serum-starved for 48 h (p<0.05 for both vs. negative control siRNA). A404 cells also demonstrated significant knockdown: 66.3% in untreated cells, 57.6% in 24 h RA-treated cells, and 70.1% at 48 h of RA-treatment (p<0.05 for all vs. negative control siRNA).

**Figure 10:**
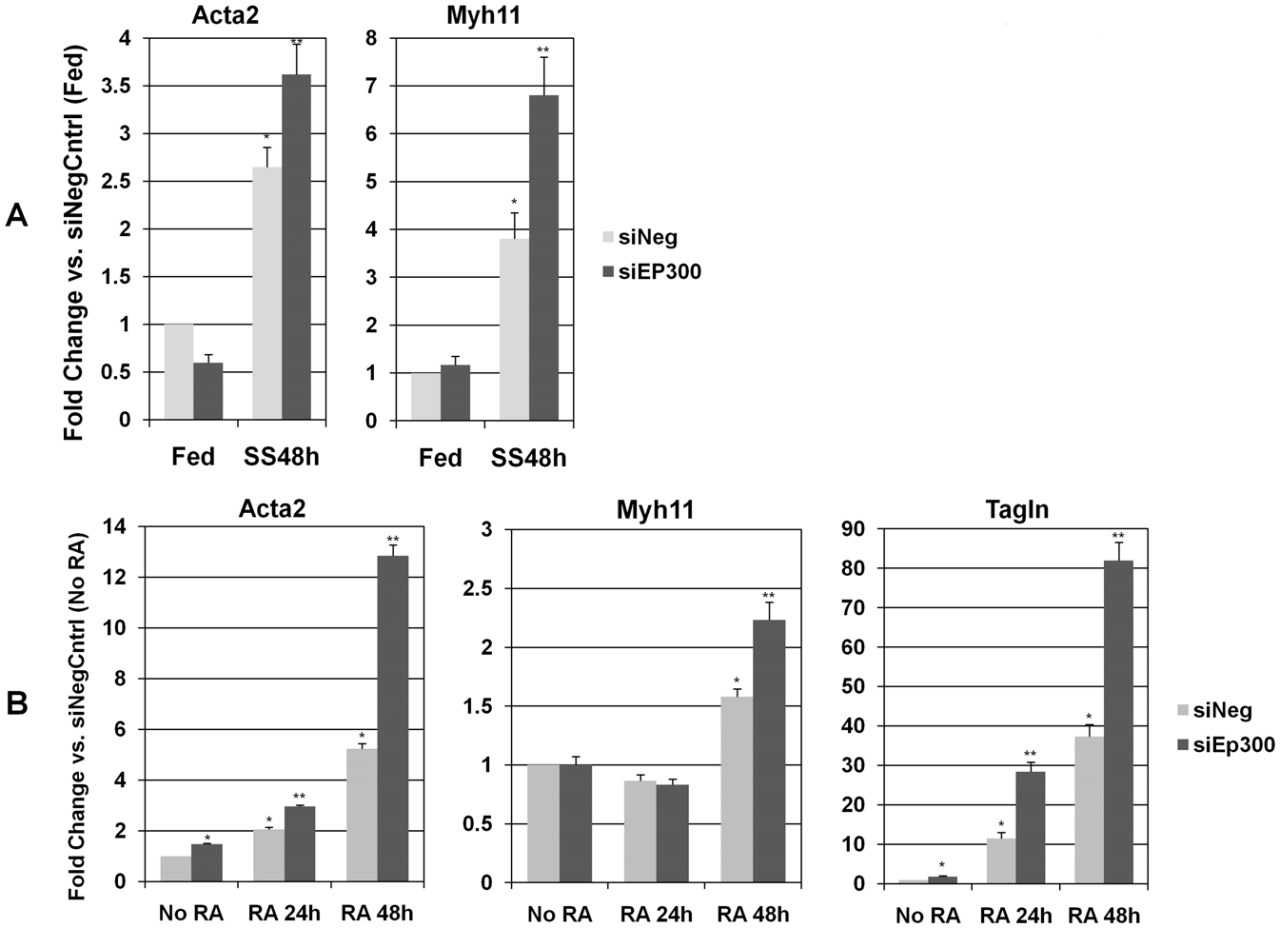
p300 siRNA knockdown and human SMC differentiation markers. **A**. Human CASMC were transfected with either scrambled negative control siRNA (siNeg) or with a transcript targeting EP300 (siEP300), and then either serum fed (Fed) or serum starved for 48 hours (SS48). Resulting qRT-PCR expression values were normalized to control (Fed cells transfected with siNeg). *  =  significantly (p<0.05) different from control. **  =  significantly (p<0.05) different from control and from siNeg time-matched controls. **B**. A404 cells were transfected with either scrambled negative control siRNA (siNeg) or with a transcript targeting Ep300 (siEp300), and then treated with retinoic acid for 24 (RA 24 h) or 48 hours (RA 48 h), or left untreated (No RA). Resulting qRT-PCR expression values were normalized to control (No RA cells, transfected with siNeg). *  =  significantly (p<0.05) different from control. **  =  significantly (p<0.05) different from control and from siNeg time-matched controls.

Serum-starvation in human SMCs transfected with negative control siRNA led to the expected increases in ACTA2 and MYH11 expression. However, cells transfected with siEP300 showed significantly higher expression of both markers after 48 hours of serum-starvation (p<0.05) (**Figure 10a** **)**.

A404 cells displayed similar behavior, with expression levels of SMC differentiation markers Acta2, Myh11 and Tagln incrementally increasing with RA-treatment over time, despite transfection with negative control siRNA. As seen in previous studies, rises in Acta2 and Tagln were already significant at 24 hours, while the increase in Myh11 was delayed. As with the human SMCs, marker expression levels increased significantly beyond negative control siRNA-transfected samples by 48 hours of RA-treatment (p<0.05 for all) (**Figure 10b**).

## Discussion

### Epigenetic regulation is a key determinant of SMC phenotypic state

SMC phenotypic change is a varied and complex process. Evidence has been steadily accruing that epigenetic regulation is a vital element in the determination of SMC differentiation state, particularly in the areas of histone acetylation and methylation.

Just as critical to SMC plasticity is the removal of inhibitory/chromatin compacting complexes from SMC-marker gene promoters, such as certain HDACs and other factors such as Klf4 [4]. These mechanisms already have established involvement in skeletal and cardiac muscle differentiation and hypertrophy [15], [38]. Further, it is necessary for SMC differentiation that SRF (a ubiquitous protein capable of activating transcription for many gene subclasses) be specifically denied to growth and proliferation genes, and that those genes remain silenced while the cells are quiescent.

Various histone methyl-lysine patterns are able to distinguish SMCs from non-SMCs [5]. Recruitment of SRF, and in particular the activating SRF/myocardin complex, to conserved CArG boxes in SMC-specific gene promoters has been associated with acetylated H3 and H4, dimethylated H3K4, and methylated H3K79 [4], [20]–[23]. A study from Lockman et al. found that a histone demethylase (Jmjda1) bound all three myocardin family members, and when overexpressed in 10T1/2 cells decreased mono- and di-methyl H3K9 while stimulating the transgelin and SM α-actin promoters [39].

### Major chromatin remodeling families undergo regulation with SMC differentiation

This study demonstrates that widespread regulation of chromatin modifying and remodeling genes takes place during SMC differentiation *in vitro*. At the maximum level observed (96 h of retinoic acid treatment), over 60% of all chromatin remodeling genes identified on the array showed significant changes in transcription from control, dramatically increasing in the second 48 h of treatment (from 17.6%). While numbers of positively and negatively regulated genes started at similar levels, by far the majority of this increase was driven by downregulated genes. As would be expected with such sweeping changes in expression, numerous classes of epigenetic regulators were represented, including DNA methyltransferases, histone methyltransferases, and others. A few highlights are presented below.

One interesting finding was the downregulation of chromobox homologs *Cbx1* and *Cbx3*. The products of these genes (HP1beta and HP1gamma) recognize tri-methylated H3K9 and mediate silencing through conversion to heterochromatin [40], [41]. Lockman et al. suggests that H3K9 demethylation at SMC-marker gene promoters may trigger conversion to euchromatin [39].

Prmt1 acts as an H4R3 arginine methyltransferase, a modification which facilitates p300-mediated histone acetylation [42], and showed mild down-regulation. A homologous gene, Prmt2, binds to the retinoic acid receptors RARα and RXRα [43], [44]. It interacts with a HAT, steroid receptor coactivator 1 (SRC-1), and might relate to RA-mediating triggering of A404 differentiation, as it is upregulated at the earliest time point and then remains unchanged.

Vire et al. [45] showed that the silencing pathways of the Polycomb group and DNA methyltransferase systems are mechanically linked. Our data imply that relief from Dnmt3a- and Dnmt3b-associated DNA methylation may be essential to preventing HDAC recruitment to SMC-specific genes during differentiation via methyl-CpG-binding proteins such as Mecp2 and Mbd2. Somewhat surprisingly, these latter two genes showed upregulation in our model, possibly reflecting a compensatory response to DNMT repression or increased protein degradation, or a redirecting of the silencing machinery toward growth and proliferation genes that could be targeted by Dnmt1 and Dnmt2 (both upregulated late in this study).

### HDAC inhibition promotes SMC differentiation: seeking the key regulators

Histone deacetylases are divided into three classes. Class I HDACs (1–3, 8) are similar to yeast RPD3 and localize to the nucleus. Class II HDACs (4–7, 9–10) resemble yeast HDA1 and are found in both nucleus and cytoplasm, while Class III HDACs are NAD-dependent enzymes similar to yeast SIR2 proteins [46]. These proteins inhibit transcription through removal of key acetyl groups. In addition to their effects on histones, some HDACs are also believed to exert effects in the cytosol through protein deacetylation. For example, HDAC8 reportedly associates with the smooth muscle actin cytoskeleton and may regulate the contractile capacity of smooth muscle cells [47].

Relief from Class II HDAC inhibition is necessary for differentiation of both skeletal and cardiac myocytes, and evidence exists for a similar role in SMCs [48]. Global HDAC suppression with trichostatin A inhibits SMC proliferation, accelerates differentiation in P19 cells, and stimulates acetylation at the transgelin locus in fibroblasts [21], [27], [49]. Qiu et al. overexpressed HDACs 1–6, and found that all of them suppressed transactivation of transgelin by Smad3 and myocardin in their cell systems [50]. Another study, however, showed that Class II HDACs 4 and 5 suppressed the ability of myocardin to activate SM α-actin and transgelin, while Class I HDACs 1 and 3 had no effect [23]. Notably, they also observed that A7r5 SMCs expressed HDACs1–2, and 4–7, but not HDAC3 or 9.

We found that A404 cells, in contrast, expressed both *Hdac3* and *9*. However, *Hdac3* was suppressed with differentiation while *Hdac9* showed progressive upregulation over time. *Hdac6* essentially disappeared with SMC differentiation. Loss of marker expression SMCs in Klf4 over-expression assays has been associated with the appearance of the H4 deacetylase HDAC2 [4], but in A404 cells *Hdac2* showed minimal change in expression during differentiation. Differences between the various experimental models might explain some of these results. We did identify a notable drop in *Hdac5* transcription with advancing differentiation, consistent with relief of suppression of myocardin. Further, we showed that inhibition of HDACs with trichostatin A accelerated A404 SMC differentiation. It remains to be established which HDACs play exactly which roles in regulating SMC plasticity.

### HAT activity of p300 plays a key role in SMC differentiation: evidence for an activated subpopulation

Previous research has offered hints that acetylation of SMC-specific promoter loci is crucial for differentiation, and evidence suggests that p300 is involved in accomplishing this [20]. Studies have identified acetyl-H3K9, -H3K14 and -H4 as distinguishing marks in SMCs (vs. non-SMCs), although it is notable that while myocardin increased acetylation of H3K9 it did not increase H4Ac, implying a separate activation step [4]. Myocardin requires SRF to activate SMC genes, and SRF has been reported to associate with the p300-related HAT CBP during c-Fos activation [51], however p300 is able to enhance myocardin independently of SRF association [23]. Further, p300 interacts with the SMC differentiation-promoting transcription factor GATA6, and the combination activates the SM-MHC promoter [24]. The ability of myocardin to activate SMC genes is enhanced by p300, which binds to its transcriptional activation domain in a separate location from HDAC5 [23].

The paralogues CBP and p300 are present in limiting amounts in mammalian cells, and signaling pathways may regulate transcription based on their ability to compete for these factors [52]–[55]. While CBP and p300 are known as HATs they may also act as transcriptional co-factors, and additionally may regulate via acetylation of non-histone proteins [14]. Numerous signaling pathways utilize these factors. Using PathwayAssist we identified 162 different proteins which have been shown to interact with p300, of which 130 were present on our microarray. In concert with the large scale transcriptional regulation of chromatin remodeling genes which occurred during the A404 time course, over half of the available p300 interactome showed significant changes. It therefore seems highly probable that global alterations in p300-based signaling accompany SMC differentiation.

Given the numerous functions of p300, it is unclear which are essential for SMC differentiation, although histone acetylation and co-activation of myocardin are supported by the evidence presented above. Our experiments show that direct chemical inhibition of p300 HAT activity substantially decreases (but does not completely arrest) SMC differentiation. Histone acetylation of H3K9, H4, and H3K14 may therefore be attributable to p300. However, interactions between p300 and myocardin might occur independently of acetyltransferase activity.

Further, alternative HATs might also be involved. While several HATs (including CBP) showed significant downregulation with SMC differentiation (**Figure 2**), Myst3 showed progressive upregulation throughout. Little is known about this factor at this time apart from its involvement in monocytic leukemia and hematopoietic stem cells [56]. Additionally the HAT PCAF was likely partially inhibited by the concentration of Lys-CoA-TAT that we employed, making our results not completely p300-specific.

Several studies have indicated that activity of CBP and p300 may depend on their phosphorylation state and be regulated by retinoic acid receptors, and that signaling may trigger the formation of an activated p300 subpopulation with increased differentiation-gene specificity [54], [57]–[61]. During retinoic acid-induced differentiation of F9 cells, p300 (but not CBP) protein levels decreased during differentiation due to increased degradation by the ubiquitin-proteasome pathway. This was accompanied by a significant increase in per molecule HAT activity, and specifically with protein kinase A-mediated phosphorylation of p300 [61].

We examined this process in A404 SMC differentiation and observed similar behavior. After treatment with retinoic acid, p300 protein levels in A404 cells progressively decreased despite only minimal changes in p300 transcription, with most of the change occurring early. The same was observed in a model of human SMC re-differentiation. Further, siRNA knockdown of p300 expression in both models accelerated SMC differentiation, suggesting that the observed decrease in p300 levels may trigger SMC differentiation.

As mentioned above, p300 is central to numerous transcriptional activation pathways, including those of proliferation and growth, and is present in limiting amounts among competing signaling processes. Thus, in SMCs undergoing differentiation, a decrease in p300 protein levels accompanied by activating covalent modifications could cause migration of the factor from growth-based pathways to promoters for myocardin and other SMC differentiation-specific genes.

More studies are needed to further elucidate this model. For example, CBP may substitute for p300 in supporting cell maintenance processes during SMC differentiation. Unlike skeletal and cardiac muscle, SMCs maintain their phenotypic plasticity. Microenvironmental triggers must therefore exist which are capable of dynamically altering the epigenetic environment [5]. Given its large interactome and key role in modulating chromatin and transcription factors, p300 presents an opportune fulcrum for regulation of SMC phenotypic modulation.
